# Supplementary material for: Inferring the Regulatory Network of miRNAs on Terpene Trilactone Biosynthesis Affected by Environmental Conditions
Source: Int J Mol Sci. 2023 Nov 30;24(23):17002. doi: 10.3390/ijms242317002 (PMC10707241; doi:10.3390/ijms242317002)
Supplement: Supplementary file 1 [file ijms-24-17002-s001.zip › ijms-2654492-supplementary.pdf]

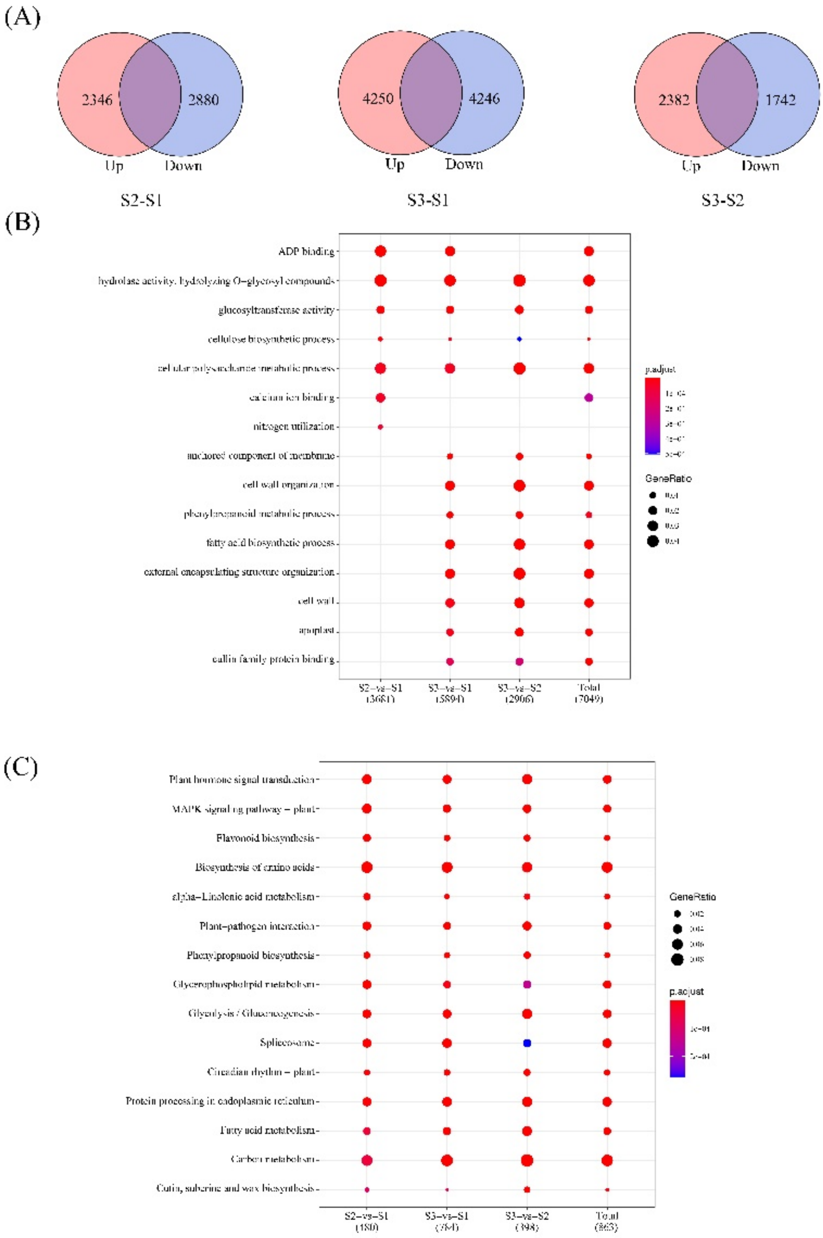

Figure S1. Differential expression genes (DEGs) analysis (A). The gene ontology (B) and KEGG pathway (C) of DEGs

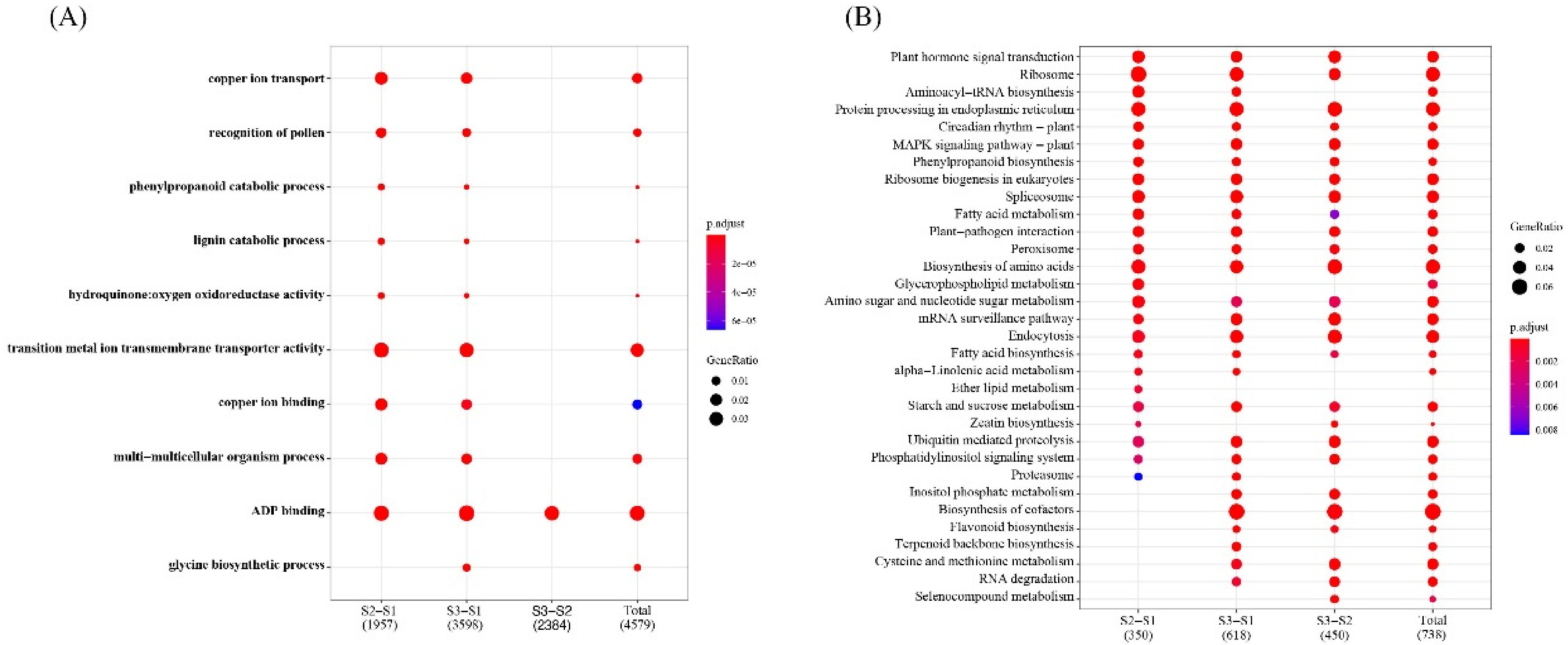

Figure S2. The gene ontology (A) and KEGG pathway (B) of the target genes of differentially expressed miRNAs (DEMs)

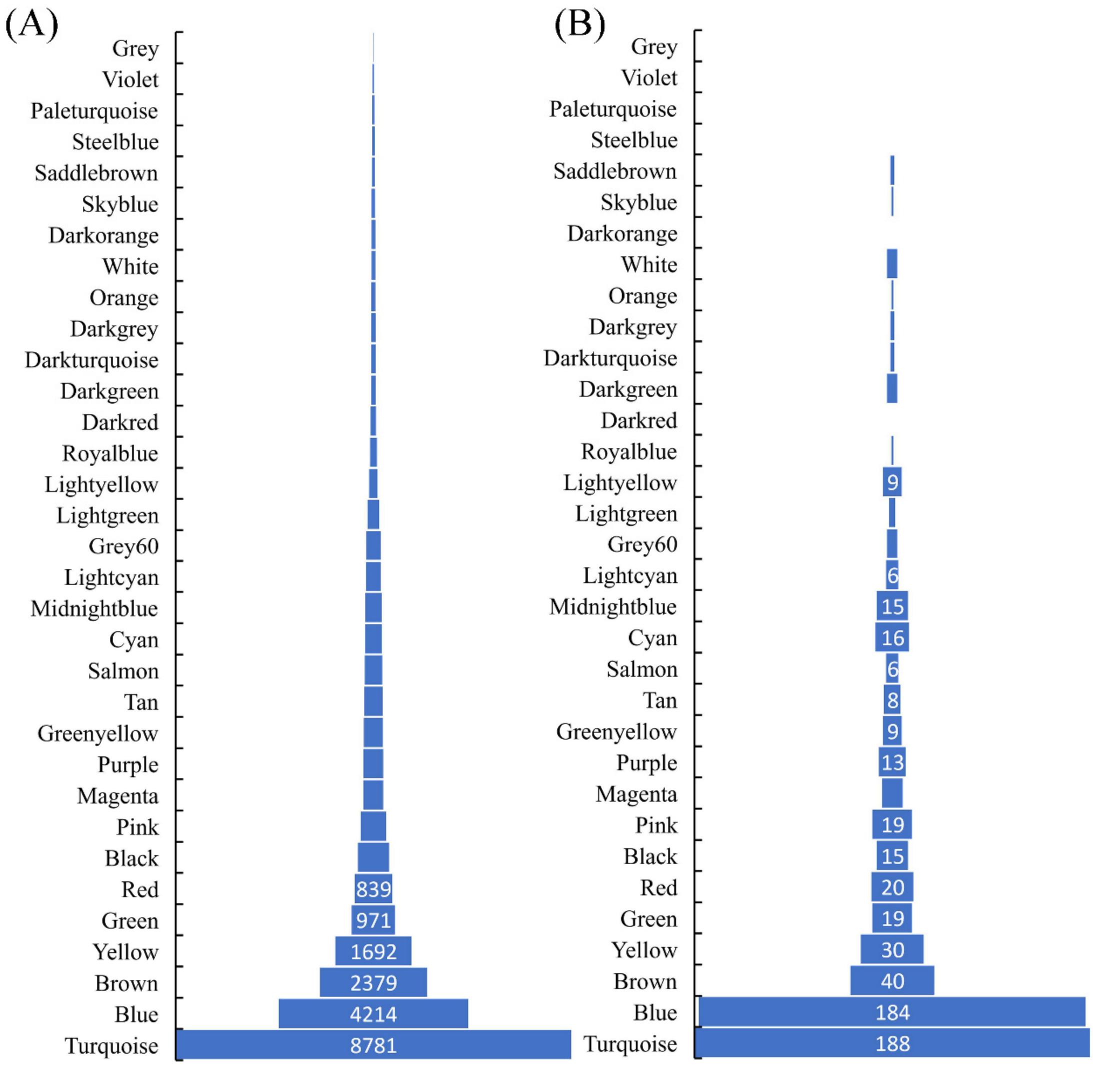

Figure S3. The number of gene (A) and transcription factor (B) in each module based on co-expression network analysis

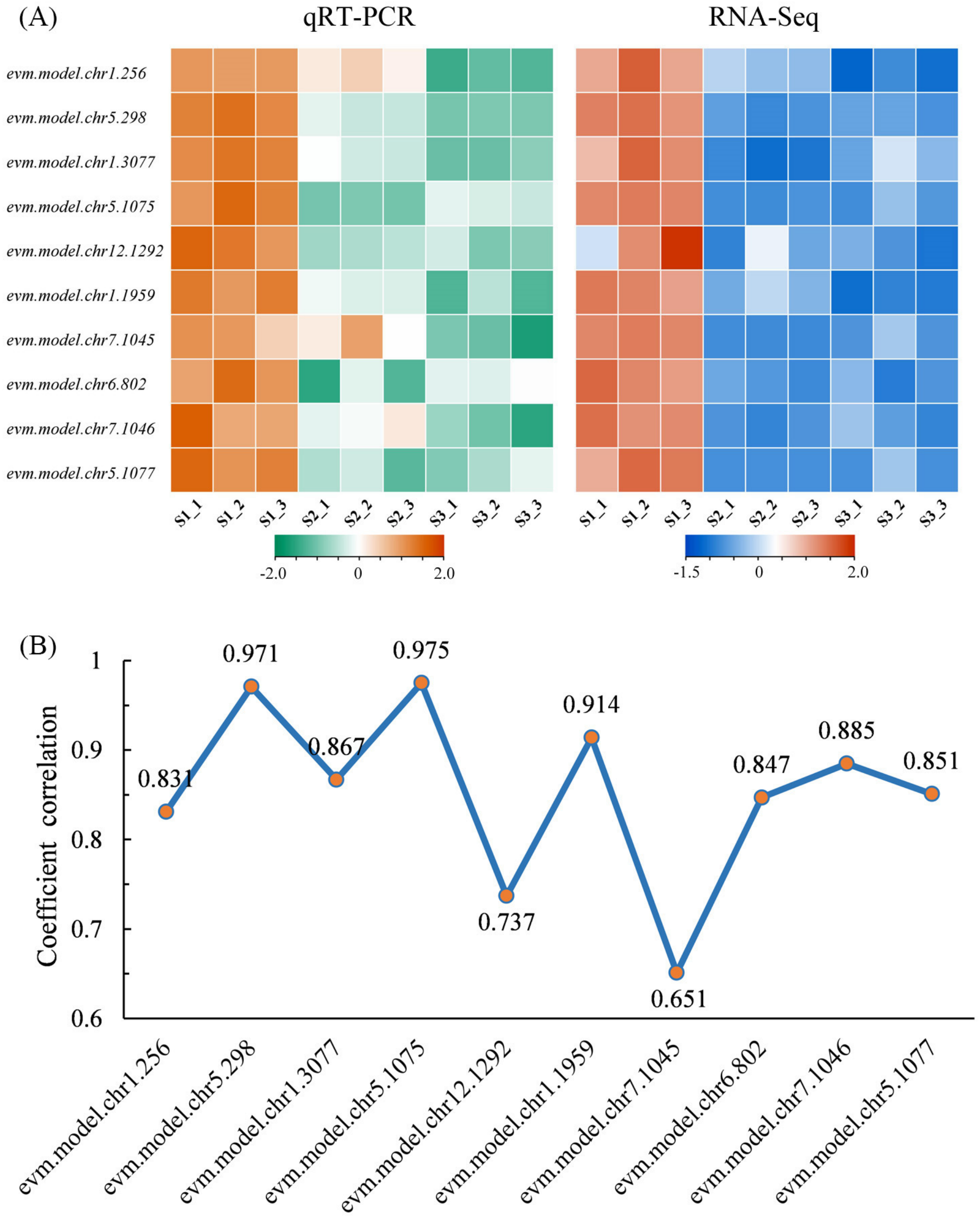

Figure S4. The qRT-PCR analysis of ten TTL-related genes (A) and correlation analysis with expression patterns based RNA sequencing (B)

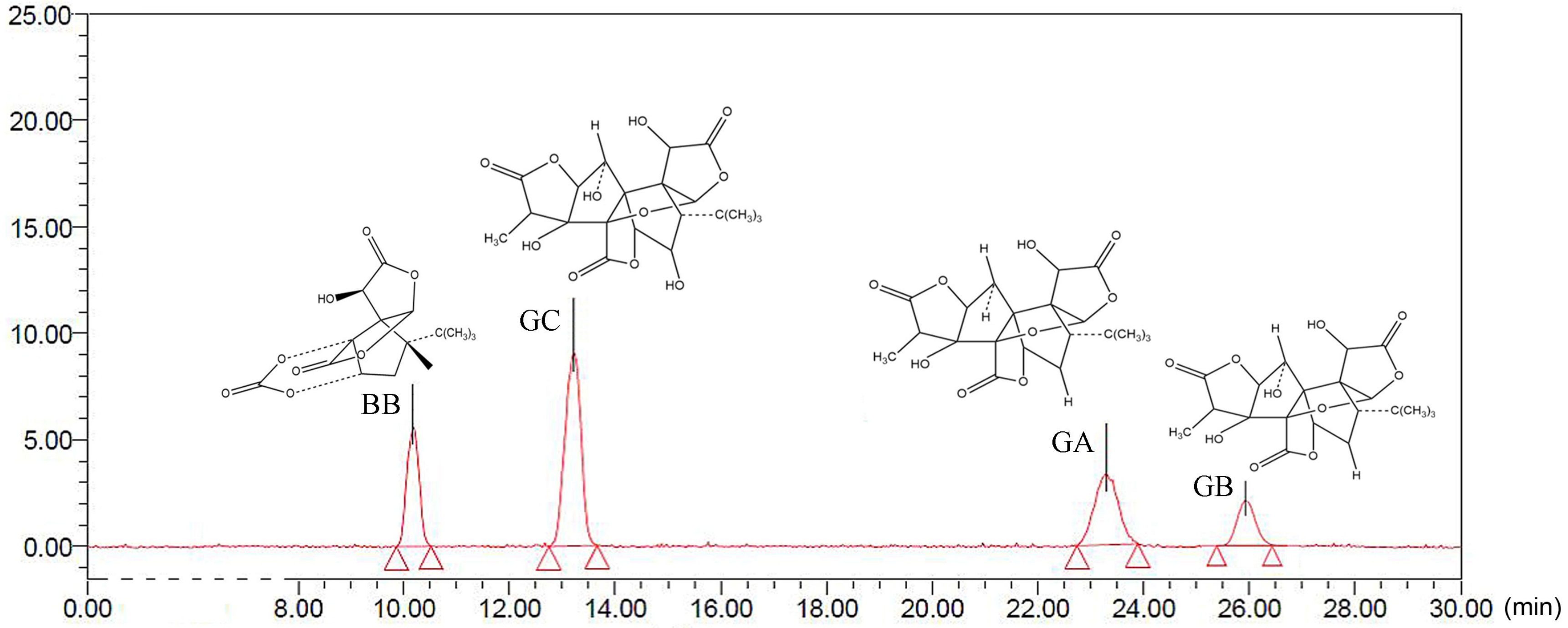

Figure S5. The HPLC chromatograms of the four standards (ginkgolide A, B, C, and bilobalide)

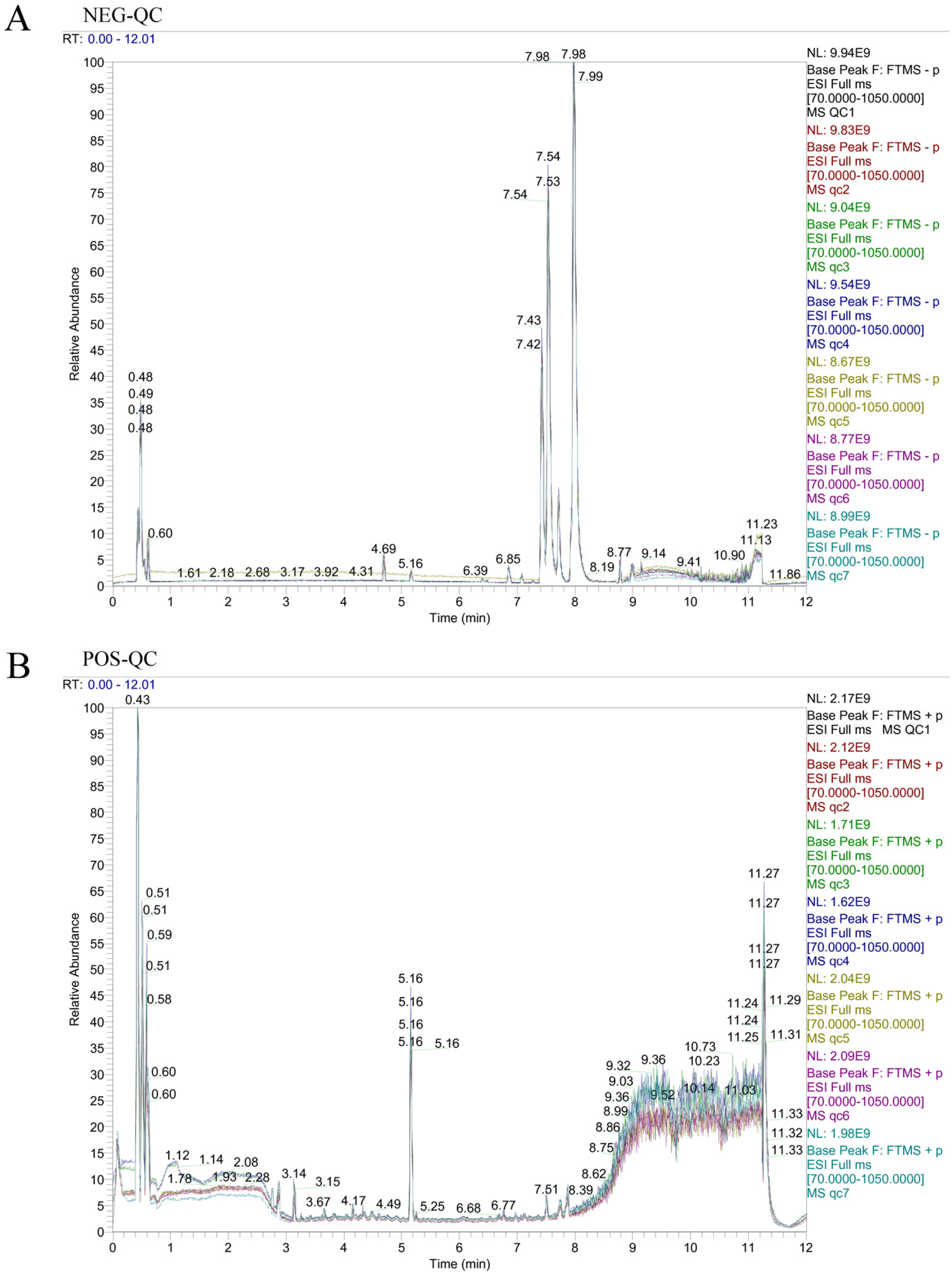

Figure S6. The mass spectrum of the QC samples with negative ion mode (A) and positive ion mode (B)
